# Large Biliary Calculus

**Published:** 1846-07

**Authors:** 


*Large Biliary Calculus.*—The person who passed it was a female aged 78 years, of a large corpulent frame, and a biliary temperament. She had symptoms of jaundice on the first of May, 1844. She was attended by a neighboring physician till June 6th, when I was called in. Her skin and every part of her body was suffused with bile, and of a dark yellow color. She complained of pain about the præcordia and right hypogastrium, with total loss of appetite, accelerated pulse, white tongue, thirst, diarrhœa, ash-colored stools, &c.

As she had been thoroughly mercurialised, I put her on the use of a strong alkaline solution, and large doses of the extract of conium, diet of eggs, &c.

By the 17th she was no better; and as symptoms of phlogosis were still manifest, I concluded there must be inflammation of the common duct, &c. I bled her freely, and applied a large blister over the region of the duct, and put her on the use of oxalic acid, half a grain in a cup of cold water every three hours daily.

Under this treatment she improved steadily, so that by the 25th her stools became bilious, and her appetite returned. In a few days she seemed quite well.

She remained free from the disease till the first of October following, when it returned and became fully established. On the 22d she commenced the use of the oxalic acid, on which she placed great reliance. She had taken it about ten days without any relief. Sitting on her chamber at that time, her stools being thin she heard something fall in the vessel like a stone. She rose up and inspected the stool, and there she found the calculus I have sent you.

Her husband washed it off, and undertook to break it with a hammer; but finding it so hard and *curious* he desisted, and laid it up for me. Her jaundice soon disappeared, and she has enjoyed excellent health ever since.

It was originally uniform on its surface, and about as large as an ordinary nutmeg, and of much the same color, but rather more ovate. That part of the outer layer which is absent, was broken off by the hammer in the gentleman's attempt to break it down.

What agency the oxalic acid had in relieving the disease in the first instance, or removing the gall-stone in the last, your readers will of course, judge for themselves.

The calculus weighs now ʒiss; and I presume, before any part was broken off, it must have weighed ʒij.—*Ibid.*

